# Agomelatine decreased cocaine-induced locomotor activity in rats; a dose-time response study

**DOI:** 10.3389/fphar.2026.1897531

**Published:** 2026-08-28

**Authors:** Susana Barbosa-Mendez, Alberto Salazar-Juárez

**Affiliations:** Laboratorio de Neurofarmacología Conductual, Microcirugía y Terapéutica Experimental. Subdirección de Investigaciones Clínicas, Instituto Nacional de Psiquiatría, Ciudad de México, Mexico

**Keywords:** 5-HT2C receptors, agomelatine, cocaine, drug addiction, locomotor sensitization, MT1/2 receptors, pharmacotherapy

## Abstract

**Introduction:**

Agomelatine (Valdoxa) is a melatonin-like antidepressant that has been shown to decrease cocaine-induced locomotor activity and dopamine release when administered at 10 mg/kg for 10 days. While major depressive disorder is typically treated with an initial dose of 25 mg/day, titrated to 50 mg/day, such dosing is unlikely for cocaine use disorder (CUD). This is due to the long-term nature of CUD treatment, the hepatotoxicity associated with high and prolonged agomelatine doses, and the increased risk of liver disease in CUD patients.

**Methods:**

Given these considerations, this study aimed to determine an effective agomelatine dosing schedule for CUD by evaluating various doses (5, 10, or 40 mg/kg) and administration durations (0, 10, or 30 days) in a locomotor sensitization model.

**Results:**

The study’s results showed that: agomelatine doses of 10 mg/kg or higher significantly decreased long-term cocaine-induced locomotor activity during the expression phase. This effect was observed regardless of whether agomelatine was administered on the first day (day 0) or after 10 or 30 days of cocaine withdrawal. Specifically, 10 mg/kg of agomelatine given for 10 days during cocaine withdrawal attenuated long-term locomotor activity induced by various cocaine doses (5, 10, or 20 mg/kg) during the expression phase. Additionally, a 30 mg/kg dose of agomelatine administered for 10 days decreased locomotor activity induced by binge-pattern cocaine administration.

**Discussion:**

Collectively, these findings suggest that a dosage of 10 mg/kg of agomelatine administered for 10 consecutive days effectively attenuates the long-term behavioral effects of cocaine.

## 1 Introduction

Cocaine use disorder (CUD) is a significant public health concern linked to various health issues (Shorter and Kosten, 2011). While numerous therapeutic approaches have demonstrated efficacy in studies (Liu, 2021), no drug has yet received FDA approval as a safe and effective treatment for CUD (Buchholz and Saxon, 2019).

Preclinical research highlights the crucial role of various neurotransmitter systems in modulating cocaine’s reinforcing effects (Bubar et al., 2006; Bubar and Cunningham, 2008). These studies suggest that 5-HT, NE, and MT receptors are key modulators of the midbrain dopaminergic (DA) system, which is fundamental for the development of psychostimulant locomotor effects (Fletcher et al., 2002; Vanderschuren et al., 2003; Onaolapo and Onaolapo, 2018). Furthermore, the activation of single or multiple NE, 5-HT, and MT receptor subtypes could offer new targets for pharmacological CUD treatments (Barbosa-Méndez and Salazar-Juárez, 2024a).

Rodent studies support this by showing that administering antagonists of α_1_ NE, 5-HT_2A_, 5-HT_2B_, or 5-HT_3_ receptors, or agonists of α_2_ NE, 5-HT_1A_, or 5-HT_2C_ receptors, attenuated cocaine-induced locomotor sensitization and conditioned place preference (Jiménez-River et al., 2006; King et al., 2000; Przegaliñski and Filip, 1997; Zayara et al., 2011), cocaine self-administration (Nic Dhonnchadha et al., 2009; Pockros-Burgess et al., 2014), and increases in extracellular dopamine levels caused by cocaine (Auclair et al., 2004; Cathala et al., 2015). Conversely, agonists of 5-HT or 5-HT_2A_ receptors, or antagonists of 5-HT_2C_ receptors, increased cocaine-induced locomotor sensitization (Zhang et al., 2016; Poc et al., 2011).

In humans, the situation is more complex. Studies evaluating antidepressant drugs that target 5-HT receptors (either singly or in combination) have yielded contradictory results. Some studies indicate these drugs attenuate cocaine’s reinforcing effects (Khouzam et al., 1995; Nanni-Alvarado et al., 2022; Walsh et al., 1994), while others report no change (Afshar et al., 2012; Schmitz et al., 2001; Duggal, 2007; Okada et al., 2020; Tapp et al., 2006).

Separately, studies have shown that MT_1_ and MT_2_ receptors are involved in modulating cocaine’s reinforcing effects (Takahashi et al., 2017). These receptors are expressed in the ventral tegmental area (VTA) and in the dopaminergic terminals of the nucleus accumbens (Nacc) (Uz et al., 2005; Imbesi et al., 2009), both critical for inducing and expressing locomotor sensitization (Vanderschuren and Pierce, 2010). Melatonin, an MT_1_ and MT_2_ receptor agonist, has been shown to decrease cocaine-induced locomotor activity (Kurtuncu et al., 2004; Barbosa-Méndez and Salazar-Juárez, 2020), reduce the motivation to seek cocaine (Takahashi et al., 2017), and mitigate cocaine and alcohol relapse-like behaviors in rats (Takahashi et al., 2017; Vengeliene et al., 2015). It also decreased cocaine-induced locomotor sensitization, intracellular dopamine levels, and cocaine-induced conditioned place preference.

These findings suggest that a drug incorporating antagonism or agonism of NE, 5-HT, or MT receptors, whether targeting a single receptor or multiple receptors simultaneously, could potentially reduce cocaine-induced reinforcing and psychomotor effects.

Agomelatine (Valdoxa), a melatonin-like antidepressant, is approved for treating major depressive disorder (MDD) (Gorwood et al., 2021). It has also shown efficacy in reducing anxiety and improving sleep quality in patients with MDD (Guo Y. H. et al., 2023; Wang et al., 2020). Agomelatine exerts its therapeutic effects by antagonizing 5HT_2B_ and 5HT_2C_ serotonergic receptors and acting as an agonist of MT_1_ and MT_2_ melatonin receptors (Bourin et al., 2004; Millan et al., 2005; San and Arranz, 2008).

Clinical studies have explored various agomelatine dosing schedules, differing in dose and administration duration, to reduce acute and chronic symptoms of MDD (Gorwood et al., 2021) and other psychiatric disorders (Li et al., 2024; Shokrani et al., 2023).

These studies have reported that, at the start of treatment, agomelatine doses generally range from 10 to 25 mg/day, and in some very specific cases, the dose may be increased to 50 mg/day.

These doses are generally administered for a period of 8–12 weeks (Ju et al., 2025; Kennedy et al., 2018; Barquera et al., 2025; Guo P. et al., 2023; Arango et al., 2025). In preclinical studies, the picture is similar, with various dosing regimens also being used. Doses range from 4–10 to 40–80 mg/kg; these doses are administered for periods of 10 days to 5 or 14 weeks (Abdelmissih et al., 2024; Papp et al., 2006; Rossetti et al., 2022; Tchekalarova et al., 2016; Barbosa-Méndez et al., 2023).

Regarding substance use disorder (CUD), different dosing schedules have also been reported (Barbosa-Méndez et al., 2023; De and Grasing, 2023; Fathi et al., 2021). For instance, a 10 mg/kg dose administered for 10 days attenuated cocaine-induced psychomotor and neurochemical effects in Wistar rats (Barbosa-Méndez et al., 2023). Similar findings were reported by De et al. (2023), who observed that a 10 mg/kg dose for 19 days decreased the reinstatement of morphine self-administration. They also noted that a 32 mg/kg/day dose generated adverse effects, primarily characterized by decreased posture (De and Grasing, 2023). In contrast, Fathi et al. (2021) reported that a 50 mg/kg dose administered for 20 days effectively decreased anxiety- and depression-like behaviors and attenuated memory impairment during alcohol withdrawal in a rat model of alcoholism (Fathi et al., 2021).

Collectively, these results suggest that while there is a consensus regarding dosing schedules for MDD treatment in humans, an effective dosing schedule for other psychiatric disorders, such as CUD, is still lacking.

Although preclinical studies support agomelatine’s efficacy in attenuating the psychomotor and reinforcing effects of illicit drugs, its potential as an effective therapeutic option for CUD must consider that, as double-blind clinical trials have shown, the therapeutic effect of antidepressant or anxiolytic drugs in humans largely depends on the specific dosing schedule used (i.e., dose and duration of treatment (Huzarska et al., 2006). Therefore, the objective of this study was to evaluate different doses (5, 10, or 40 mg/kg) of agomelatine and different administration times (0, 10, or 30 days) in the locomotor sensitization model to determine an effective dosing schedule for the treatment of CUD.

In this study, we utilized locomotor sensitization to investigate agomelatine’s effect on the development and establishment of cocaine use disorder. Behavioral sensitization has been linked to long-term neuroadaptive brain changes, increasing drug salience, locomotor activity, and compulsive drug-seeking behaviors (Vanderschuren and Pierce, 2010). These changes are crucial factors in the escalation of drug use and contribute to relapses in drug use disorders (Robinson and Berridge, 1993).

## 2 Materials and methods

### 2.1 Animals

We used male *Wistar* rats weighing 250–280 g at the beginning of the study. The Institutional Animal Vivarium provided animals. Rats were housed in groups of four in standard plastic rodent cages (57 cm × 35 cm × 20 cm) in a colony room maintained at constant temperature (21 °C ± 2 °C) and humidity (40%–50%) on a 12-h/12-h light/dark cycle (lights on at 7:00 a.m.). The animals had continuous access to rodent chow pellets and water, except during the experimental sessions. We previously reported that melatonin significantly decreased cocaine-induced locomotor activity in the light phase (Barbosa-Méndez and Salazar-Juárez, 2020) thus, all the experiments took place during the light phase of the light/dark cycle (between 9:00 a.m. and 3:00 p.m.). The Institutional Animal Care Committee and Bioethics Committee (Protocol # CEI/C/015/2022) approved the procedures in strict compliance with the Guide for the Care and Use of Laboratory Animals published by the National Institutes of Health (NIH) and reported in accordance with ARRIVE guidelines.

### 2.2 Drugs

The preparation of the drugs was carried out according to previously reported studies (Barbosa-Méndez et al., 2023). Cocaine hydrochloride was kindly donated by the Mexican government under strict regulatory controls. All the drugs used in experimental animals were kept under official surveillance (COFEPRIS- LC-0004-2003). Agomelatine (VALDOXA, Servier) was purchased after obtaining the required regulatory permission, as per official guidelines (COFEPRIS-2016, Mexico). Cocaine hydrochloride was dissolved in sterile saline solution (0.9% NaCl, Sigma Aldrich). Agomelatine (VALDOXA, Servier) 25 mg tablets were pulverized, and the resulting powder was dissolved in saline solution. The solutions were freshly prepared before intraperitoneal (i.p.) administration to the animals. During the experiments, the solutions were maintained at 4 °C. Saline (0.9% NaCl) was used as a control in all experiments. To determine whether agomelatine could prevent the effects of cocaine, it was administered 30 min before cocaine or saline. The treatments were injected into a volume of 1 mL/kg body weight.

#### 2.2.1 Dose selection

The agomelatine doses (5, 10, or 40 mg/kg) were determined by previous reports showing that these doses of agomelatine are a safe, tolerated, and effective dose to decrease anxiety and depressive-like behavior in rats (Li et al., 2024; Tchekalarova et al., 2016). Preclinical studies have reported the administration of agomelatine doses of 40, 50, or even 80 mg/kg for a period of 10 days to 14 weeks. These studies did not report any significant adverse effects (Abdelmissih et al., 2024; Papp et al., 2006; Rossetti et al., 2022). The doses of cocaine were chosen based on previous studies showing that a dose of 10 or 20 mg/kg of cocaine generates a robust increase in cocaine-induced locomotor activity and behavioral sensitization (Bar et al., 2017) but could not generate seizures.

### 2.3 Behavioral sensitization procedure

#### 2.3.1 Apparatus

In this study, locomotor activity was evaluated in transparent plexiglass activity chambers (50 cm × 50 cm × 30 cm) set attached to a PC, according to a previously reported protocol. Each of them was surrounded by a 16 × 16 array of photocell beams located 3 cm from the floor surface to scan locomotor activity (OMNIALVA, Instruments, Mexico). Interruptions of the photo-beams were automatically quantified with OABiomed software (1.1) and then analyzed. Locomotor activity was defined as consecutive beam breaks (Salazar-Juárez et al., 2016) (OMNIALVA, Mexico).

#### 2.3.2 Procedure

Locomotor activity was assessed using a previously reported experimental protocol (Salazar-Juárez et al., 2016). This began 1) with the habituation of the animals to the activity chambers which took place for three 30-minute sessions; 2) Subsequently, the animals were randomly assigned to different pharmacological treatment groups. 3) At the start of the behavioral recording session, rats were injected with the treatment that was previously assigned; 4) Afterwards, the rats immediately returned to that activity chamber and locomotor activity was recorded for 30 min; and 5) the rats were returned to their home cages after each experimental session had been completed (Salazar-Juárez et al., 2016).

#### 2.3.3 Euthanasia

The animals were euthanized in a separate room by means of an overdose of pentobarbital sodium (Sedal-Vet, 65 mg/mL) (Barbosa-Méndez and Salazar-Juárez, 2024b). The sacrifice was carried out by a method accepted and approved by the Official Mexican Standard NOM-062-ZOO-1999 (1999).

### 2.4 Experimental procedures

A pilot study was conducted at the start of the research to ensure ethical compliance regarding animal care, particularly animal reduction. This pilot study revealed no significant differences in baseline locomotor activity among the three SAL + AGO groups (5, 10, and 40 mg). It also confirmed that administering a fixed dose of agomelatine (10 mg/kg) for up to 30 days did not alter baseline locomotor activity. Given these findings, and to adhere to the principle of animal reduction, a single SAL + AGO control group was used in the main study, as no significant differences were observed between SAL + AGO groups subjected to varying doses and administration times.

Consequently, the study utilized 192 male Wistar rats, distributed among four main experiments. For Experiments 1 and 2, 48 animals were used, further divided into six experimental groups (n = 8). Experiment 3 involved 64 animals, assigned to eight groups (n = 8). For Experiment 4, 32 animals were used in four experimental groups (n = 8). Each experimental group received a distinct pharmacological treatment.

The protocol for Experiments 1, 2, and 3 comprised several phases:

- Phase I: Induction of cocaine-induced locomotor sensitization.
- Phase II: Determining whether long-term administration of different treatments (varying doses and administration times of agomelatine) during the cocaine-withdrawal phase decreases the expression of cocaine-induced locomotor sensitization.
- Phase III: Assessing agomelatine’s effect on the expression of cocaine sensitization and its ability to reduce drug relapses.
- Phase IV: Determining if the agomelatine-induced decrease in the expression of cocaine sensitization is independent of the continuous presence of agomelatine.

#### 2.4.1 Experiment 1: agomelatine in different doses modified the expression of cocaine sensitization

**FIGURE 1:**
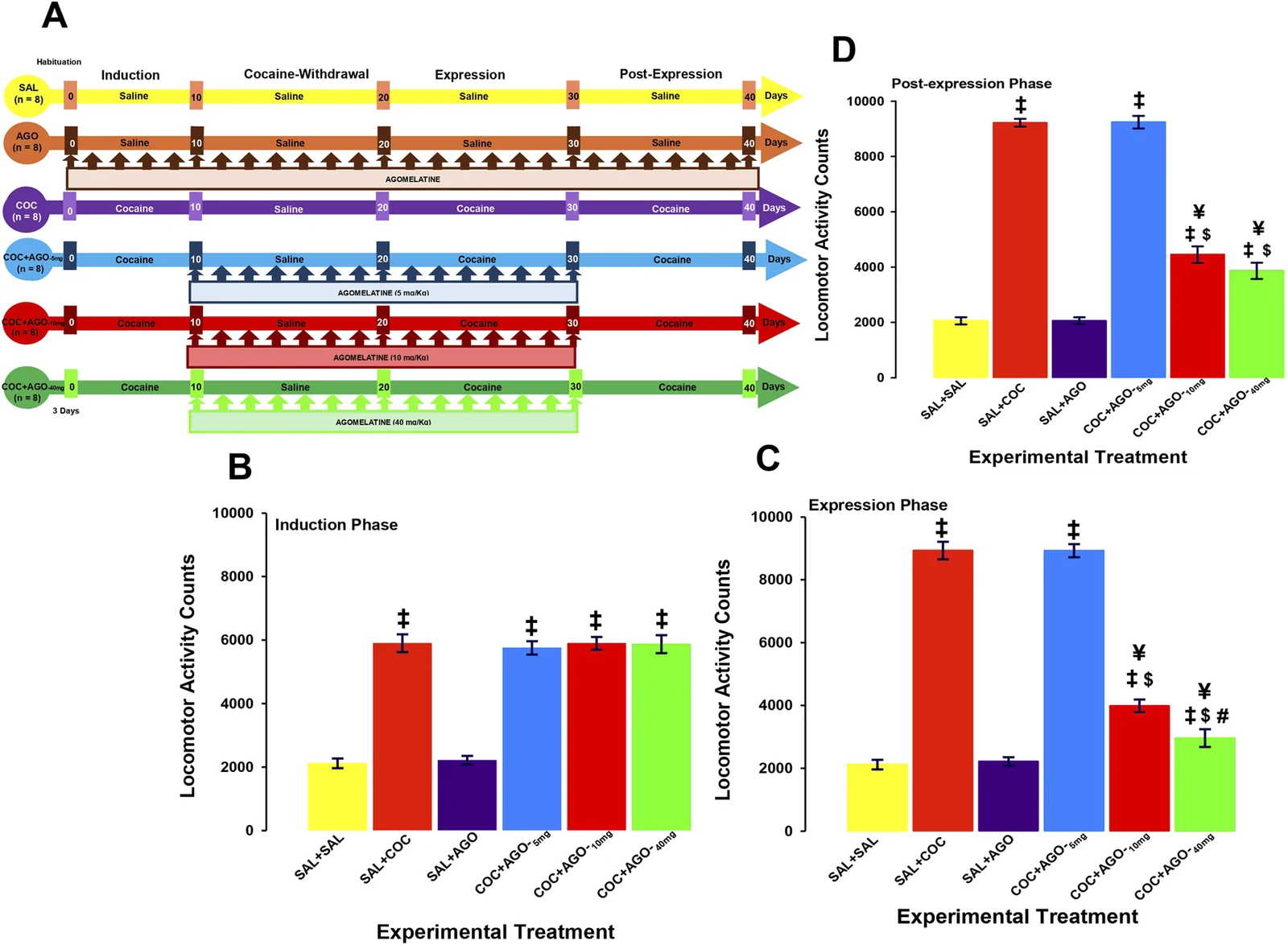
**(A)** Timeline of Experiment 1. **(B)** Mean ± S.E.M. locomotor activity by group (n = 8 animals per group) during induction, **(C)** expression, and **(D)** post-expression of locomotor sensitization. ^‡^ *p* < 0.01 Significant effect of cocaine treatment on locomotor activity compared to the SAL + SAL and the SAL + AGO groups. ^¥^ *p* < 0.01 Significant effect of agomelatine treatment on locomotor activity compared to the SAL + COC group. ^$^ *p* < 0.01 Significant effect of 10 mg/kg of agomelatine treatment on locomotor activity, compared to the COC + AGO_−5__mg_ and COC + AGO_−40__mg_ groups, ^#^ *p* < 0.01 Significant effect of 40 mg/kg of agomelatine treatment on locomotor activity, compared to the COC + AGO_−5__mg_ and COC + AGO_−10__mg_ groups, determined by a three-way ANOVA followed by a Tukey’s test. Inset shows the effect of different doses of agomelatine on locomotor activity during **(B)** induction, **(C)** expression, and **(D)** post-expression phase.

The experiment comprised four 10-day phases: cocaine-induction (Phase I), cocaine-withdrawal (Phase II), cocaine-expression (Phase III), and post-expression (Phase IV) (Figure 1A).

Throughout all four phases, the SAL + SAL group received daily saline solution (0.9% NaCl, i.p.), and the SAL + AGO group received daily agomelatine (10 mg/kg, i.p.). The cocaine group (SAL + COC) received daily cocaine (10 mg/kg, i.p.) during the induction, expression, and post-expression phases. During the cocaine-withdrawal phase, cocaine was withdrawn, and the animals received only daily saline solution.

The cocaine + agomelatine groups (COC + AGO_−5mg_, COC + AGO_−10mg_, and COC + AGO_−40mg_) received daily cocaine (10 mg/kg, i.p.) during the induction and post-expression phases. During the cocaine-withdrawal phase, groups received agomelatine (5, 10, or 40 mg/kg, i.p., depending on the group) 30 min before saline. In the cocaine-expression phase, rats received agomelatine 30 min before cocaine (10 mg/kg, i.p.). Locomotor activity was recorded for 30 min after each daily administration.

#### 2.4.2 Experiment 2: the changes in the expression of cocaine sensitization depended on the duration of the agomelatine treatment

This experiment evaluated the effect of agomelatine, following the protocol described in Experiment 1. For the cocaine plus agomelatine treatment groups, the cocaine-withdrawal phase varied in duration (0, 1, 10, or 30 days).

**FIGURE 2:**
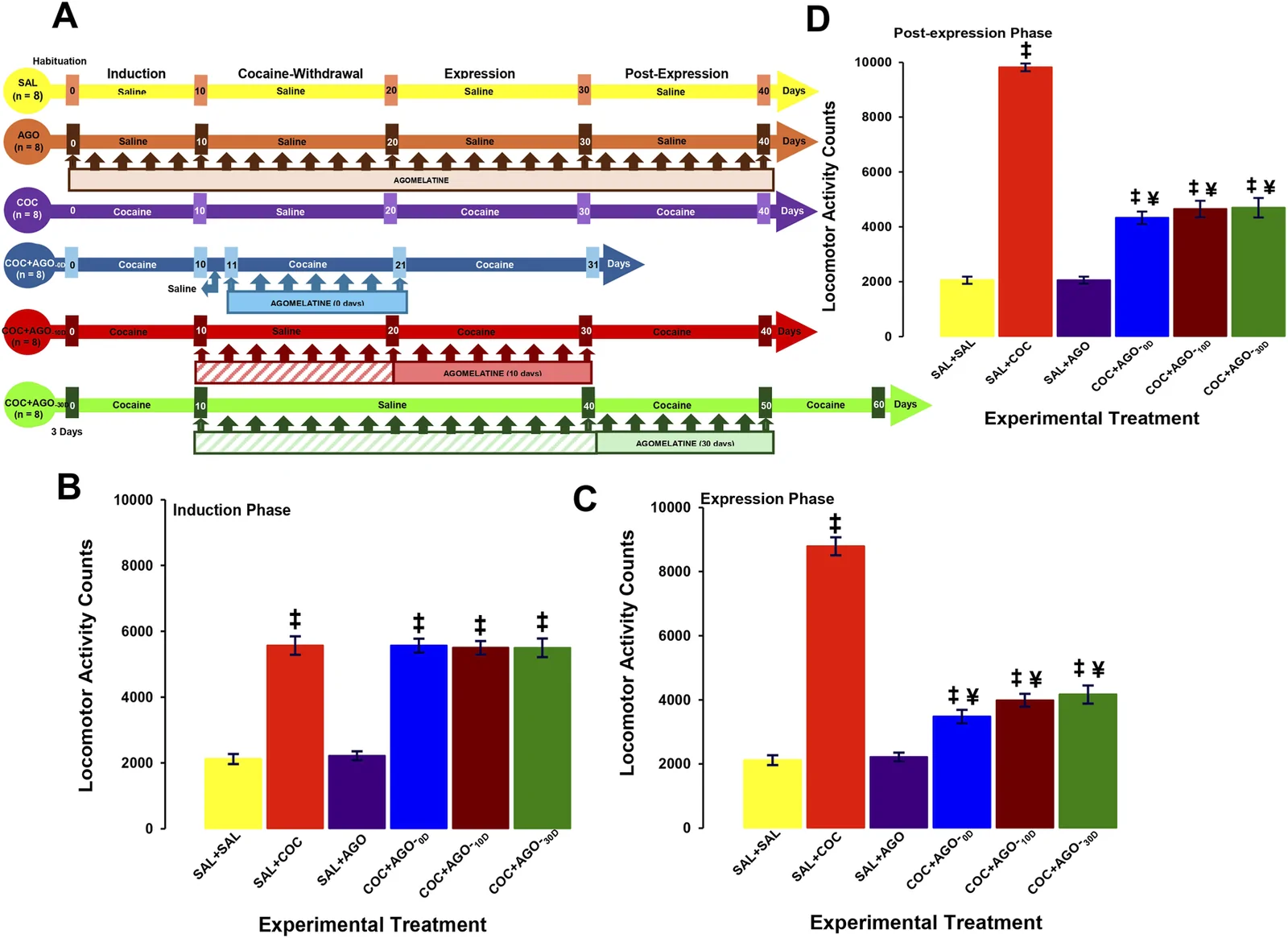
**(A)** Timeline of Experiment 2. **(B)** Mean ± S.E.M. locomotor activity by group (n = 8 animals per group) during induction, **(C)** expression, and **(D)** post-expression of locomotor sensitization. ^‡^ *p* < 0.01 Significant effect of cocaine treatment on locomotor activity compared to the SAL + SAL and the SAL + AGO groups. ^¥^ *p* < 0.01 Significant effect of agomelatine treatment on locomotor activity compared to the SAL + COC group, determined by a three-way ANOVA followed by a Tukey’s test. Inset shows the effect of different doses of agomelatine on locomotor activity during **(B)** induction, **(C)** expression, and **(D)** post-expression phase.

The SAL + SAL, SAL + AGO, and SAL + COC groups received treatments as detailed in Experiment 1 (Figure 2A). The cocaine + agomelatine groups (COC + AGO_−0D_, COC + AGO_−10D_, and COC + AGO_−30D_) were administered cocaine daily during the induction and post-expression phases. During both the cocaine withdrawal and expression phases, rats received a fixed dose of agomelatine (10 mg/kg, i.p.) 30 min before the subsequent administration of either saline (during withdrawal) or cocaine (during expression; 10 mg/kg, i.p.). Locomotor activity was recorded for 30 min after each administration.

#### 2.4.3 Experiment 3: agomelatine modified the expression of cocaine sensitization

**FIGURE 3:**
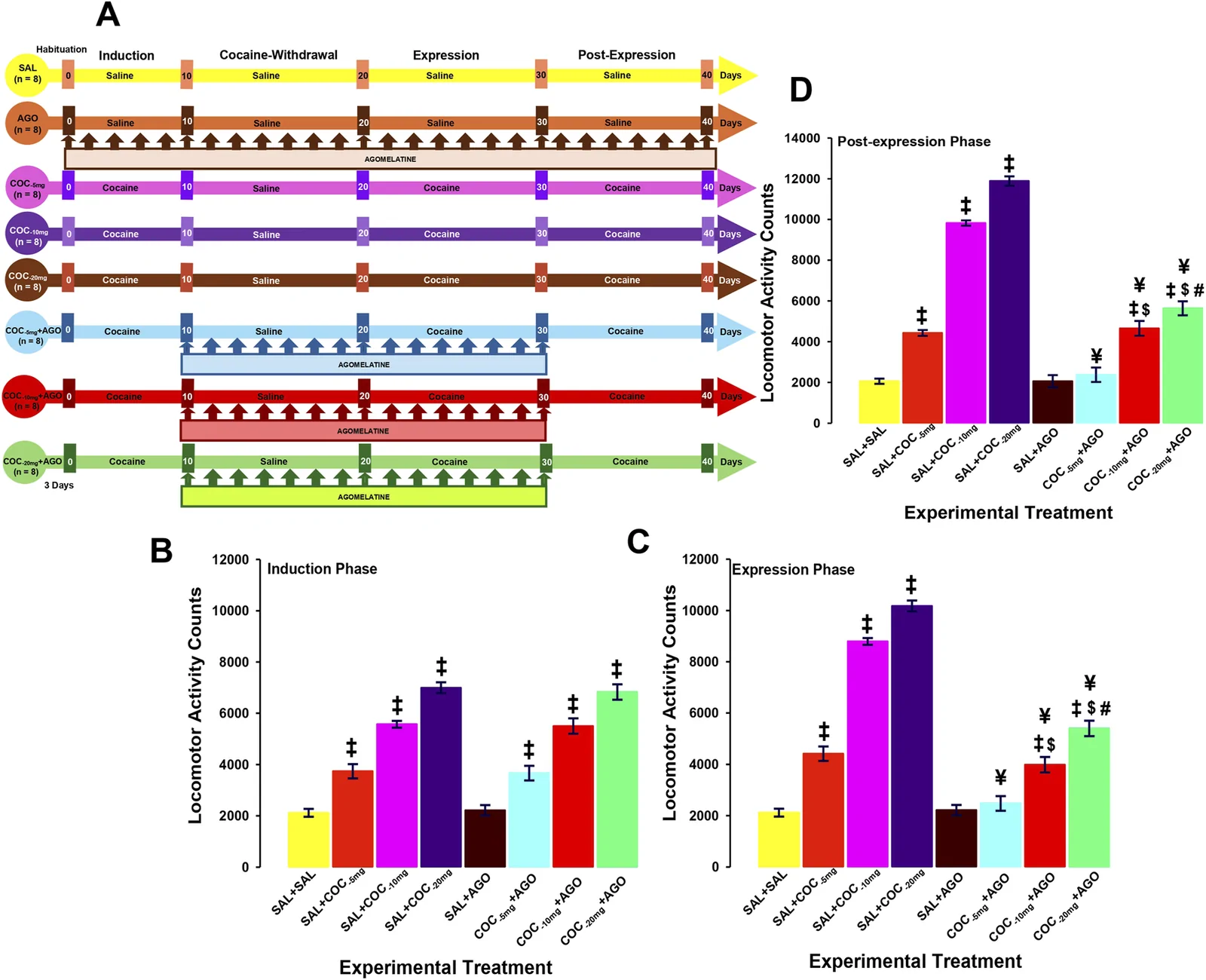
**(A)** Timeline of Experiment 3. Mean ± S.E.M. locomotor activity by group (n = 8 animals per group) during **(B)** induction, **(C)** expression, and **(D)** post-expression of behavior sensitization. ^‡^p < 0.01 Significant effect of cocaine treatment on locomotor activity compared to the SAL + SAL and the SAL +AGO groups. ^¥^p < 0.01 Significant effect of agomelatine treatment on locomotor activity compared to the SAL + COC group. ^$^p < 0.01 Significant effect of 10 mg/Kg of cocaine treatment on locomotor activity, compared to COC-5mg + AGO and the COC-20mg + AGO groups, ^#^p < 0.01 Significant effect of 20 mg/Kg of cocaine treatment on locomotor activity, compared to the COC-5mg + AGO and the COC-10mg + AGO groups, determined by a three-way ANOVA followed by a Tukey’s test.

The experiment followed the protocol described in Experiment 1. The SAL + SAL and SAL + AGO groups received treatments as detailed in Experiment 1 (Figure 3A).

The cocaine groups (SAL + COC_−5mg_, SAL + COC_−10mg_, and SAL + COC_−20mg_) received cocaine daily at doses of 5, 10, and 20 mg/kg, i.p., respectively, during the induction, expression, and post-expression phases. During the cocaine-withdrawal phase, these groups received daily saline.

For the cocaine + agomelatine groups (COC_−5mg_ + AGO, COC_−10mg_ + AGO, and COC_−20mg_ + AGO), cocaine was administered daily at 5, 10, and 20 mg/kg, i.p., respectively, during the induction and post-expression phases. During the cocaine-withdrawal phase, these animals received agomelatine (10 mg/kg, i.p.) 30 min prior to daily saline administration. In the cocaine-expression phase, agomelatine (10 mg/kg, i.p.) was given 30 min before the respective cocaine doses (5, 10, or 20 mg/kg, i.p.).

#### 2.4.4 Experiment 4: agomelatine suppresses the binge cocaine administration

**FIGURE 4:**
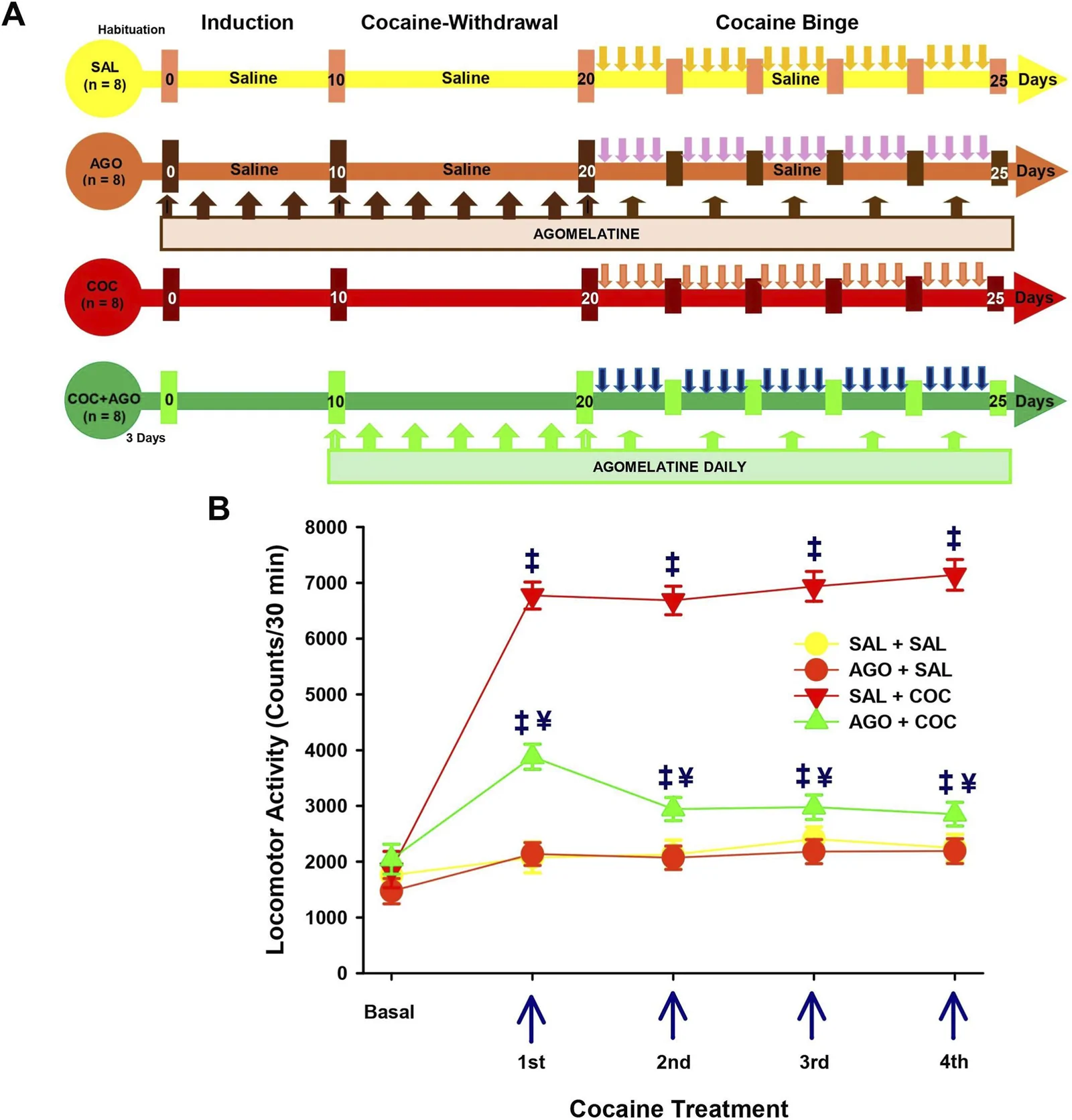
**(A)** Timeline of Experiment 4. Each point represents mean ± S.E.M. (n = 8 animals per group) locomotor activity recorded for 30 minutes after each injection. **(B)** Locomotor activity in rats, following “binge” pattern cocaine administration ^‡^p < 0.01 Significant effect of cocaine treatment on locomotor activity compared to the SAL + SAL and the SAL +AGO groups. ^¥^p < 0.01 Significant effect of agomelatine treatment on locomotor activity compared to the SAL + COC group, determined by a two-way repeated measures ANOVA followed by a Tukey’s test.

Experiment 4 included three phases: Phase I, cocaine induction (10 days); Phase II, cocaine withdrawal (10 days); and Phase III, the cocaine binge phase (5 days) (Figure 4A).

During Phases I and II, the SAL + SAL, SAL + AGO, SAL + COC, and COC + AGO groups received the treatments described in Experiment 1. In the cocaine binge phase (Phase III), SAL + SAL, SAL + AGO, and SAL + COC groups received four daily administrations of sterile saline solution (0.9% NaCl, i.p.), agomelatine (30 mg/kg, i.p.), and cocaine (10 mg/kg, i.p.), respectively. These treatments were administered every 3 h for 5 consecutive days, with the first administration taking place at 10:00 a.m.

For the COC + AGO group, agomelatine (10 mg/kg, i.p.) was administered 30 min before the beginning of each daily experimental session, followed by cocaine according to the aforementioned binge pattern. After the treatments, locomotor activity was recorded for each subject for 30 min.

### 2.5 Statistical analysis

Data were expressed as mean ± S.E.M. Locomotor activity was measured by counting beam breaks during the testing session. During the pilot study, the results for basal locomotor activity in each group were analyzed with a one-way analysis of variance (ANOVA) followed by a Tukey’s HSD test for *post hoc* comparisons.

In Experiment 1–3, a three-way analysis of variance (ANOVA) was used, with treatment (saline and agomelatine), groups (saline and cocaine), and agomelatine doses (5, 10 or 40 mg/kg), or agomelatine administration duration (0, 10, and 30 days), or cocaine doses (5, 10 or 20 mg/kg) as the between-subjects factor, following a Tukey’s *post hoc* test.

To analyze the effect of the agomelatine on the locomotor sensitization, a four-way ANOVA was used, with groups, treatment, agomelatine or cocaine doses, or agomelatine administration duration and phase (induction and expression) as the between-subject factors.

A two-way repeated measure ANOVA with the treatment as the within-subjects factor and group as the between-subjects factor, and minutes as the repeated measure time factor was performed to determine the differences in the locomotor responses during the cocaine binge phase. The statistical significance level was set at *p* < 0.05. The statistical analysis was performed with SPSS Statistics 21 (IBM).

## 3 Results

The pilot study demonstrated that agomelatine administered at different doses and times did not alter baseline locomotor activity (See results in Supplementary Material).

### 3.1 Experiment 1: agomelatine in different doses modified the expression of cocaine sensitization

As shown in Figure 1B, during the induction phase (three-way ANOVA; in the groups by treatment by doses interaction; F = (1, 96) 0.89, *p* = 0.91; ƞ^2^ = 0.002), the cocaine dose (10 mg/kg) given to the SAL + COC, the COC + AGO_−5mg_, the COC + AGO_−10mg_, and COC + AGO_−40mg_ groups resulted in a significant increase in locomotor activity compared to the SAL + SAL (*p* < 0.001) and the SAL + AGO (*p* < 0.001) groups (Figure 1B).

In the expression phase, treatment with 10 mg/kg of cocaine failed to increase locomotor activity in rats previously dosed with 10 or 40 mg/kg of agomelatine. The COC + AGO_−10mg_ and the COC + AGO_−40mg_ groups showed significant differences (three-way ANOVA; in the groups by treatment by doses interaction; F = (1, 96) 52.790, *p* < 0.001; ƞ^2^ = 0.55) in mean locomotor activity, compared to the SAL + SAL (*p* < 0.001), the SAL + AGO (*p* < 0.001), the SAL + COC (*p* < 0.001), and the COC + AGO_−5mg_ (*p* < 0.001) groups. The Tukey’s test revealed significant differences between the COC + AGO_−10mg_ and the COC + AGO_−40mg_ (*p* < 0.001) groups. Additionally, in this experimental phase, cocaine given to animals treated with 5 mg/kg agomelatine significantly increased (*p* < 0.001) locomotor activity (Figure 1C).

Upon withdrawal of agomelatine, the rats treated with 10 or 40 mg/kg agomelatine showed significant differences (three-way ANOVA; in the groups by treatment by doses interaction; F = (1,96) 34.959 *p* < 0.001; ƞ^2^ = 0.45) compared to the SAL + SAL (*p* < 0.001), the SAL + AGO (*p* < 0.001), the COC + COC (*p* < 0.001), and the COC + AGO_−5mg_ (*p* < 0.001) groups (Figure 1D). These results suggested that a dose of 10 mg/kg agomelatine was enough to produce a persistent attenuation (≈50%) in cocaine-induced locomotor activity, whereas a daily dosing with 5 mg failed to decrease cocaine evoked hyperlocomotion. In addition, the results suggest that a dose of 40 mg/kg could not improve the effect produced by a dose of 10 mg/kg agomelatine.

#### 3.1.1 Locomotor sensitization

To determine whether the administration of different doses of agomelatine altered the cocaine locomotor sensitization (Figures 3A–D, 4A–C), four-way ANOVA (F (1.192) = 27.086, *p* < 0.001; ƞ^2^ = 0.24) found differences in the group × treatment × doses × phase interaction. Tukey’s test found significant differences in the cocaine-induced locomotor activity shown during the induction phase compared to that shown in the expression phase in the SAL + COC (*p* < 0.001), COC + AGO_−5mg_ (*p* < 0.001), COC + AGO_−10mg_ (*p* < 0.002), and the COC + AGO_−40mg_ (*p* < 0.001) groups.

### 3.2 Experiment 2: the changes in the expression of cocaine sensitization depended on the duration of the agomelatine treatment

Cocaine administered daily to the SAL + COC, the COC + AGO_−0D_, the COC + AGO_−10D_, and the COC + AGO_−30D_ groups significantly increased locomotor activity during the induction phase (three-way ANOVA; in the groups by treatment by time interaction; F = (1, 96) 0.07, *p* = 0.99; ƞ^2^ = 0.001), compared to the SAL + SAL (*p* < 0.001) and the SAL + AGO (*p* < 0.001) groups (Figure 2B).

In contrast, during the expression phase, a fixed dose of agomelatine (10 mg/kg) for 0, 10 or 30 consecutive days resulted in significant differences (three-way ANOVA; in the groups by treatment by time interaction; F = (1, 96) 7.79, *p* < 0.001; ƞ^2^ = 0.15) in cocaine-induced locomotor activity, compared to the SAL + SAL (*p* < 0.001), the SAL + AGO (*p* < 0.001), and the SAL + COC (*p* < 0.001) groups (Figure 2C).

Upon withdrawal of agomelatine, the animals treated for 0, 10, or 30 days with 10 mg/kg agomelatine showed significant differences (three-way ANOVA; in the groups by treatment by time interaction; F = (1,96) 18.29 *p* < 0.001; ƞ^2^ = 0.30), compared to those in the SAL + SAL (*p* < 0.001), the SAL + AGO (*p* < 0.001), and the SAL + COC (*p* < 0.001) groups (Figure 2D).

These results suggest that 1) agomelatine has an immediate therapeutic effect on cocaine-induced locomotor activity, which is an advantage in the treatment of DUC, and 2) increasing the duration of agomelatine treatment, up to 30 days, did not improve the effect on cocaine-induced locomotor activity.

#### 3.2.1 Locomotor sensitization

To determine whether the administration of agomelatine, during different dosing times, altered the cocaine locomotor sensitization (Figures 3A–D; 4A–C; 5A,B), four-way ANOVA (F (1.192) = 3.32, *p* < 0.03; ƞ^2^ = 0.64) found differences in the group × treatment × time × phase interaction. Tukey’s test found significant differences in the cocaine-induced locomotor activity shown during the induction phase compared to that shown in the expression phase in the SAL + COC (*p* < 0.001), COC + AGO_−0D_ (*p* < 0.001), COC + AGO_−10D_ (*p* < 0.002), and the COC + AGO_−30D_ (*p* < 0.001) groups.

### 3.3 Experiment 3: agomelatine modified the expression of cocaine sensitization

Treatment with cocaine induces a dose-dependent increase (three-way ANOVA; in the groups by treatment by doses interaction; F = (1, 96) 0.06, *p* = 0.99; ƞ^2^ = 0.001) in the locomotor activity in the SAL + COC_−5mg_, the SAL + COC_−10mg_, the SAL + COC_−20__mg_, the COC_−5__mg_ + AGO, the COC_−10__mg_ + AGO, and the COC_−20__mg_ + AGO groups compared to the SAL + SAL (*p* < 0.001) and the SAL + AGO (*p* < 0.001) groups (Figure 3B).

Treatment with a fixed agomelatine dose (10 mg/kg) for 10 days produced a significant decrease (three-way ANOVA; in the groups by treatment by doses interaction; F = (1,96) 22.14, *p* < 0.001; ƞ^2^ = 0.34) in cocaine-induced locomotor activity during the expression phase (Figure 3C) in the COC_−5__mg_ + AGO, the COC_−10__mg_ + AGO and the COC_−20__mg_ + AGO groups, compared to the SAL + COC_−5__mg_ (*p* < 0.001), the SAL + COC_−10__mg_ (*p* < 0.001), the SAL + COC_−20__mg_ (*p* < 0.001) groups. Furthermore, the *post hoc* test revealed differences in locomotor activity displayed by the COC_−10__mg_ + AGO, and the COC_−20__mg_ + AGO groups compared to that shown by the SAL + SAL (*p* < 0.001) and SAL + AGO (*p* < 0.001) groups.

Tukey’s test found significant differences in cocaine-induced locomotor activity displayed by the COC_−5__mg_ + AGO group compared to that in COC_−10__mg_ + AGO (*p* < 0.001), and the COC_−20__mg_ + AGO (*p* < 0.001) groups; in addition, it revealed differences between the COC_−10__mg_ + AGO, and the COC_−20__mg_ + AGO (*p* < 0.001) groups (Figure 3C).

Upon withdrawal of agomelatine, different doses of cocaine given daily to animals previously treated with agomelatine showed a dose-dependent increase in locomotor activity (three-way ANOVA; in the groups by treatment by doses interaction; F = (1, 96) 56.07, *p* < 0.001; ƞ^2^ = 0.57). A Tukey’s *post hoc* test revealed significant differences between mean locomotor activity in the COC_−5__mg_ + AGO, the COC_−10__mg_ + AGO, and the COC_−20__mg_ + AGO groups, compared to the SAL + COC_−5__mg_ (*p* < 0.001), the SAL + COC_−10__mg_ (*p* < 0.001), and the SAL + COC_−20__mg_ (*p* < 0.001) groups (Figure 3D).

This suggested that a dosing regimen consisting of 10 mg/kg agomelatine for 10 consecutive days was enough to both permanently reduce the locomotor activity induced by different doses of cocaine.

#### 3.3.1 Locomotor sensitization

To determine whether the administration of agomelatine altered the locomotor sensitization induced by different doses of cocaine (Figures 3A–D; 4A–C; 5A,B), four-way ANOVA (F (1.192) = 12.605, *p* < 0.001; ƞ^2^ = 0.13) found differences in the group × treatment × doses × phase interaction. Tukey’s test found significant differences in the cocaine-induced locomotor activity shown during the induction phase compared to that shown in the expression phase in the SAL + COC_−5__mg_ (*p* < 0.002), the SAL + COC_−10__mg_ (*p* < 0.001), the SAL + COC_−20__mg_ (*p* < 0.001), the COC_−5__mg_ + AGO (*p* < 0.002), COC_−10__mg_ + AGO (*p* < 0.002), and the COC_−20__mg_ + AGO (*p* < 0.001) groups.

### 3.4 Experiment 4: agomelatine suppresses the binge cocaine administration

Agomelatine dosing significantly decreased the locomotor activity produced by a binge pattern cocaine administration (two-way repeated measures ANOVA; treatment × time interaction F = (1, 28) 539.27, *p* < 0.0001; ƞ^2^ = 0.98). Tukey’s test revealed significant differences in locomotor activity in the COC + AGO group compared to the SAL + SAL (*p* < 0.002), the SAL + AGO (*p* < 0.002), and the SAL + COC (*p* < 0.0001) groups.

These results suggested that agomelatine at 30 mg/kg for 30 days was able to significantly decrease locomotor activity induced by a binge-pattern cocaine administration.

## 4 Discussion

This study revealed four key findings: 1) Agomelatine, at doses of 10 mg/kg or higher, decreased long-term cocaine-induced locomotor activity during the expression phase of locomotor sensitization. 2) Administering agomelatine on day 0, day 10, or day 30 of cocaine withdrawal reduced long-term cocaine-induced locomotor activity during the expression phase. 3) A 10-day regimen of 10 mg/kg agomelatine during cocaine withdrawal decreased long-term locomotor activity induced by various cocaine doses (5, 10, or 20 mg/kg) during the expression phase. 4) A 10-day regimen of 30 mg/kg agomelatine decreased locomotor activity induced by binge-pattern cocaine administration.

As mentioned previously, several preclinical and clinical studies reported that the agomelatine-induced antidepressant effect was dose-dependent (Li et al., 2024; Barquera et al., 2025; Guo P. et al., 2023; Arango et al., 2025; Abdelmissih et al., 2024; Papp et al., 2006; Rossetti et al., 2022). Consistent with these findings, our study observed a dose-dependent decrease in cocaine-induced locomotor activity.

Regarding CUD, a 10 mg/kg dose of agomelatine has been reported to attenuate cocaine-induced psychomotor and neurochemical effects (Barbosa-Méndez et al., 2023) and decrease the reinstatement of morphine self-administration (De and Grasing, 2023). In contrast, Fathi et al. (2021) reported that 50 mg/kg decreased anxiety- and depression-like behaviors and attenuated memory impairment during alcohol withdrawal (Fathi et al., 2021).

Collectively, these studies suggest a dose-response relationship for agomelatine’s antidepressant effect, potentially indicating better therapeutic outcomes at higher doses. However, this must be weighed against significant safety concerns: several studies have indicated that chronic administration of agomelatine, particularly increasing the dose (e.g., from 25 to 50 mg/day) or initiating treatment with high doses (≥50 mg/day), increases the risk of hepatotoxic adverse reactions (Gahr et al., 2013; Gahr et al., 2015). Indeed, even chronic administration (3–6 months) of 25 mg/day of agomelatine has been reported to cause hepatotoxic reactions, characterized by elevated transaminases and cytolytic hepatitis (Freiesleben and Furczyk, 2015; Montastruc et al., 2014). In contrast, clinical studies evaluating the therapeutic effect of 10 mg/kg of agomelatine have reported no adverse side effects related to alterations in liver function (Gahr et al., 2013; Gahr et al., 2015).

Treating CUD is complicated by its chronic nature, requiring long-term interventions (Mateu-Mollá et al., 2025). Compounding this, cocaine use itself significantly increases the risk of developing liver dysfunction, often leading to rapidly developing clinical symptoms of hepatotoxicity (Jastrzębska and Daniel, 2023; Kwentoh et al., 2023; Zacharia and Jacob, 2024).

Given these factors—the inherent liver risk in CUD patients and the known hepatotoxic adverse reactions of higher-dose agomelatine—a dosing schedule of 25 or 50 mg for extended periods would not be safe for individuals with CUD. Instead, a 10 mg/kg dose of agomelatine administered for 10 days appears to be a promising dosing schedule for CUD treatment. This is supported by the results of this study, reports by Barbosa-Mendez et al. (2023) and De and Grasing (2023), and further by our laboratory’s findings that agomelatine doses ≤10 mg/kg do not alter spontaneous locomotor activity, balance, or motor coordination during either the light or dark period (Barbosa-Méndez and Salazar-Juarez, 2025). Nevertheless, comprehensive safety studies for both cocaine and agomelatine are still required to confirm the safety of the proposed dosing program and to establish the exact parameters that avoid major hepatotoxic effects.

Preclinical and clinical studies consistently indicate that the therapeutic effect of antidepressants is associated with treatment duration, with effects typically emerging slowly and becoming evident only after 6–8 weeks of continuous treatment (Huzarska et al., 2006). For agomelatine, specifically, clinical trials report that 8–12 weeks are required to achieve its maximum therapeutic effects (Barquera et al., 2025; Guo P. et al., 2023). However, the findings of this study contradict these established observations. We observed that 10 mg/kg of agomelatine acutely decreased cocaine-induced loco motor activity, with no significant differences in effect found between the first day, 10 days, or 30 days of administration. This acute finding is consistent with reports by Barbosa-Mendez et al. (2023), De and Grasing (2023), and Fathi et al. (2021), who administered 10 or 50 mg/kg of agomelatine for up to 20 days (Barbosa-Méndez et al., 2023; De and Grasing, 2023; Fathi et al., 2021).

This result holds significant implications. Firstly, it expands the potential use of agomelatine to earlier stages of anti-addiction therapy, such as the detoxification phase—where effective medications are currently lacking—rather than limiting its application to the withdrawal maintenance phase. Secondly, restricting the administration duration to 10 days could mitigate the risk of liver disorders associated with prolonged agomelatine use.

Therefore, our findings suggest that an effective dosing schedule could involve 10 mg/kg of agomelatine administered for 10 days. A critical question, however, remains: would this dosing schedule prove effective in scenarios common among patients with CUD, such as multiple cocaine use (a binge pattern) or exposure to toxic cocaine doses?

As mentioned previously, this dosing schedule resulted in a long-term decrease in cocaine-induced locomotor activity during locomotor sensitization. Preclinical studies have shown that agomelatine induces neuroplastic changes, primarily characterized by an increase in synaptic spine density in regions such as the hippocampus and prefrontal cortex (Lee et al., 2025; Abdelaziz et al., 2024; Demir Özkay et al., 2015); It is possible that this mechanism participates in the long-term effects induced by agomelatine; however, further studies are needed to validate this hypothesis.

Clinical reports indicate that cocaine abusers often develop tolerance to the subjective effects of intravenous cocaine, leading to increased amounts and frequency of use. This escalation frequently culminates in acute cocaine intoxication, characterized by cardiovascular complications, seizures, and even death (Ritz and George, 1993).

In this study, 10 mg/kg of agomelatine administered for 10 days decreased locomotor activity induced by 10 or 20 mg/kg of cocaine (doses considered sublethal). To our knowledge, this specific result has not been previously reported. Agomelatine likely decreased cocaine-induced hyperactivity and mitigated the effects of higher cocaine doses by binding to 5-HT_2C_ receptors, which are known to play a significant role in increased locomotor activity and the development of cocaine toxicity (Ritz and George, 1997a; Ritz and George, 1997b). Furthermore, a dose-response relationship between increasing cocaine dose and cocaine-induced dopamine release has been well-documented (Heien et al., 2005). In this context, agomelatine may also attenuate this dose-dependent dopamine release by binding to MT_1_ and MT_2_ receptors, which have been reported to decrease cocaine-induced dopamine release (Barbosa-Méndez et al., 2023). This dual mechanism would explain the reduction in locomotor activity induced by various doses of cocaine.

Patients with CUD often consume varying doses of cocaine, frequently intermittent cycles of repeated dosing or “binges,” which produce changes in mood, cocaine craving, and various cardiovascular and subjective effects (Foltin and Fischman, 1997; Ward et al., 1997a; Ward et al., 1997b).

Repeated cocaine binge administration in both rats and humans is known to cause severe toxicity, manifesting as psychosis, ataxia, convulsions, lethargy, and even death (Schlussman et al., 1998; Segal and Kuczenski, 1997).

Our current study found that 10 mg/kg of agomelatine, administered for 10 days, decreased locomotor activity induced by binge-pattern cocaine administration—a previously unreported finding. This builds upon our earlier work showing that melatonin partially reduced similar cocaine-induced locomotor activity. Together, these results suggest that sustained agonism at MT_1_ and MT_2_ receptors may diminish the effects of binge-pattern cocaine administration.

Consequently, agomelatine could offer a therapeutic advantage in patients with cocaine abuse by reducing cocaine-induced psychosis and toxicity during binge administration, potentially decreasing associated mortality. Nevertheless, controlled clinical studies are essential to fully evaluate agomelatine’s efficacy in mitigating these toxic effects.

As mentioned previously, drugs with a multi-target pharmacological profile, which simultaneously incorporate in their mechanism of action the antagonism of 5-HT_2A_ and 5-HT_2B_ receptors and the agonism of MT_1_ and MT_2_ receptors, could reduce the cocaine-induced reinforcing and psychomotor effects. Recently, we evaluated the effect of antidepressants, antipsychotics, and other drugs, which in their mechanism of action incorporate agonism and/or antagonism of 5-HT receptors (Barbosa-Méndez and Salazar-Juárez, 2024a). The results suggested that simultaneous antagonism of 5-HT_2A_, 5-HT_2B_, and 5-HT_3_ receptors and agonism of the 5-HT_2C_ receptor was the ideal pharmacological combination for the development of new multi-target drugs for the treatment of CUD (Barbosa-Méndez and Salazar-Juárez, 2024a). However, a limitation of this study was the lack of evaluation of drugs with a melatonin-like pharmacological profile.

In this study, a 10 mg/kg dose of agomelatine, administered for at least 10 days during cocaine withdrawal, effectively reduced locomotor sensitization to cocaine. This suggests that the combination of MT_1_ and MT_2_ receptor agonism and 5-HT receptor antagonism yields good results. However, this study has a limitation in that it does not directly assess addiction-related behaviors like compulsive drug seeking and use, cocaine motivation, and relapses, despite the significant role sensitization plays in long-term neuroadaptive changes leading to increased drug salience, locomotor activity, and compulsive drug-seeking and craving behaviors. Nonetheless, these findings indicate that agomelatine could complement other approved pharmacological treatments for cocaine use disorder. Still, further studies blending pharmacology and behavior are necessary to confirm its effectiveness.

## Data Availability

The original contributions presented in the study are included in the article/Supplementary Material, further inquiries can be directed to the corresponding author.
