# Supplementary material for: Agomelatine decreased cocaine-induced locomotor activity in rats; a dose-time response study
Source: Front Pharmacol. 2026 Aug 28;17:1897531. doi: 10.3389/fphar.2026.1897531 (PMC13561897; doi:10.3389/fphar.2026.1897531)
Supplement: Supplementary file 1 [file Supplementaryfile1.doc]

**AGOMELATINE DECREASED COCAINE-INDUCED LOCOMOTOR ACTIVITY IN RATS; A DOSE-TIME RESPONSE STUDY**

Susana Barbosa Méndez and Alberto Salazar-Juárez

**Running Title:** Mirtazapine attenuates anxiety and depressive-like behavior

**SUPPLEMENTARY METHODS**

**Pilot Study**

**Doses-Response**

**Induction**

**Expression**

**Post-expression**

**Pilot Study:**

**Time-Response**

**Induction**

**Expression**

**Post-expression**

**Sensitization Indices**

**Doses-Response**

**Cocaine- 1.51**

**AGO5mg- 1.04**

**AGO10mg- 0.67**

**AGO40mg- 0.50**

**Time-Response**

**Cocaine- 1.58**

**AGO0D- 0.88**

**AGO10D- 0.62**

**AGO30D- 0.75**

**Induction**

**Expression**
